# Novel members of bacterial community during a short-term chilled storage of common carp (*Cyprinus carpio*)

**DOI:** 10.1007/s12223-021-00935-4

**Published:** 2021-12-07

**Authors:** Edit Kaszab, Milán Farkas, Júlia Radó, Adrienn Micsinai, Brigitta Nyírő-Fekete, István Szabó, Balázs Kriszt, Béla Urbányi, Sándor Szoboszlay

**Affiliations:** 1 Department of Environmental Safety, Institute of Aquaculture and Environmental Safety, Hungarian University of Agriculture and Life Sciences, 1 Páter Károly, 2100 Gödöllő, Hungary; 2 Department of Molecular Ecology, Institute of Aquaculture and Environmental Safety, Hungarian University of Agriculture and Life Sciences, 1 Páter Károly, 2100 Gödöllő, Hungary; 3 Department of Environmental Toxicology, Institute of Aquaculture and Environmental Safety, Hungarian University of Agriculture and Life Sciences, 1 Páter Károly, 2100 Gödöllő, Hungary; 4 Department of Aquaculture, Institute of Aquaculture and Environmental Safety, Hungarian University of Agriculture and Life Sciences, 1 Páter Károly, 2100 Gödöllő, Hungary; 5 WESSLING Hungary Ltd, 6 Anonymus, 1045 Budapest, Hungary

## Abstract

This work aimed to identify the key members of the bacterial community growing on common carp (*Cyprinus carpio*) fillets during chilled storage with next-generation sequencing (NGS) and cultivation-dependent methods. Carp fillets were stored for 96 h at 2 °C and 6 °C with and without a vacuum package, and an additional frozen-thawed storage experiment was set for 120 days. Community profiles of the initial and stored fish samples were determined by amplicon sequencing. Conventional microbial methods were used parallelly for the enumeration and cultivation of the dominant members of the microbial community. Cultivated bacteria were identified with 16S rRNA sequencing and the MALDI-TOF MS method. Based on our results, the vacuum package greatly affected the diversity and composition of the forming microbial community, while temperature influenced the cell counts and consequently the microbiological criteria for shelf-life of the examined raw fish product. Next-generation sequencing revealed novel members of the chilled flesh microbiota such as *Vagococcus vulneris* or *Rouxiella chamberiensis* in the vacuum-packed samples. With traditional cultivation, 161 bacterial strains were isolated and identified at the species level, but the identified bacteria overlapped with only 45% of the dominant operational taxonomic units (OTUs) revealed by NGS. Next-generation sequencing is a promising and highly reliable tool recommended to reach a higher resolution of the forming microbial community of stored fish products. Knowledge of the initial microbial community of the flesh enables further optimization and development of processing and storage technology.

**Supplementary information:**

The online version contains supplementary material available at 10.1007/s12223-021-00935-4.

## Introduction

Common carp is an extremely perishable food (Li et al. 2017a); spoilage occurs rapidly leading to a limited shelf-life for carp products (Gram and Huss 1996). Spoilage of carp products is due to unstable chemical composition and microbial growth (Sterniša et al. 2016). The growing microbial community is the most common cause of biochemical changes leading to spoilage of fish products and making them unacceptable for human consumption (Arashisar et al. 2004).

The initial microbial load and the first few days of storage have a significant effect on the microbiological quality (Odeyemi et al. 2020). The initial bacterial community of stored carp is mainly composed of psychrotrophic Gram-negative bacteria from the genera *Pseudomonas*, *Moraxella*, *Acinetobacter*, *Shewanella*, and *Flavobacterium* (Gram and Huss 1996). Depending on their spoilage potential, some of these organisms may contribute to the quality changes of fish (Beaz-Hidalgo et al. 2014). The fraction of the total initial microbiota with a role in spoilage is called the specific spoilage organisms (Parlapani et al. 2014). During storage, the microbial community of the stored carp dramatically changes due to the influence of several intrinsic or extrinsic factors. Therefore, in the critical phases, such as the first few days of storage, it is important to determine the key members of the microbial community down to the species level. To identify the species of bacteria from samples, traditional cultivation methods, often merged with 16S rRNA gene sequencing, have been utilized previously (Zhang et al. 2015; Wang et al. 2017). Based on these findings, a total of 13 different genera comprised the microbial communities of fresh common carp fillets with the dominance of *Acinetobacter* species. In air-packed (AP) samples, *Pseudomonas* became dominant on day 4, while in vacuum-packed (VP) samples, *Aeromonas* constituted the largest group on day 6. During storage of VP carp fillets, H_2_S producing bacteria and especially lactic acid bacteria (LAB) counts increased slightly towards the end of the shelf life (Zhang et al. 2015).

High-resolution techniques such as next-generation sequencing (NGS) have been used recently in the microbial characterization of stored fish products. With the combination of a culture-based method and high-throughput sequencing, the predominance of *Aeromonas* and *Lactococcus* species was revealed in refrigerated common carp samples (Zhang et al. 2017a). In spoiled freeze-chilled muscle samples of common carp, *Pseudomonas* proved to be the dominant genus (Li et al. 2018). However, the resolution of these research works reached the genus level only and did not identify the key species of the stored carp fillet. Similarly, culture-independent NGS methods of crucian carp (*Carassius auratus*) showed the predominance of *Pseudomonas* during partial freezing and chilled storage (Zhang et al. 2017b), while the microbial characterization of vacuum-packed crisp grass carp (*Ctenopharyngodon idella*) revealed the dominance of *Aeromonas* and *Pseudomonas* species (Pan et al. 2018). Based on an overview of the scientific literature, we could not find any next-generation sequencing result of stored common carp microbiome determination at a species level. Similarly, no NGS results represented the European production and processing conditions of common carp. Therefore, this work aimed to follow the microbial changes in carp fillet samples originating from a Hungarian aquaculture under different storage temperatures (2 °C, 6 °C, frozen-thawed) and package (aerobic package, and vacuum package) conditions for 96 h with cultivation-dependent and cultivation-independent methods. The cultivation-dependent approach combined isolation techniques with partial 16S rRNA sequencing and MALDI-TOF MS, while next-generation sequencing was the chosen cultivation-independent technique to identify the dominant bacterial taxa.

## Materials and methods

### Sampling and packaging

**Fig. 1:**
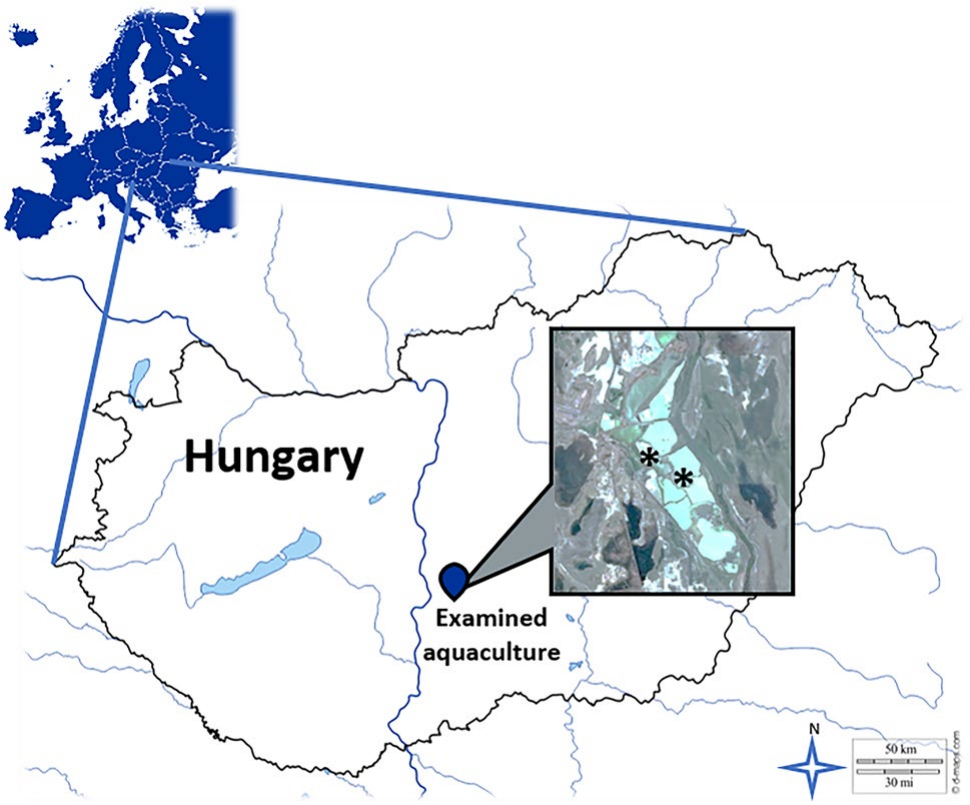
Location of the sampling site of the chosen Hungarian aquaculture. Sampling points are marked with an asterisk (original maps: d-maps.com^©^, Google Earth^®^)

The sampling site was a shallow fishpond located in the sodic area of Central Hungary with a semi-intensive (low population density) aquaculture (Fig. 1). Waterbody and sediment were sampled in 2018 autumn at two points (marked with an asterisk) representing inflow water and the waterbody of the fishpond. The microbiological state of the waterbody was revealed by the pour plate technique (MSZ EN ISO 6222 2000). Water chemistry parameters were determined by Wessling Hungary Ltd. (Budapest, Hungary) with standardized analytical methods (MSZ EN ISO 9963–1 1998; MSZ EN ISO 10304–1 2009; MSZ EN ISO 17294–2 2005). Fifteen common carp (*Cyprinus carpio*) with average body mass (2.5 ± 0.5 kg) were obtained from the fishpond in the same autumn period. Live carps were immediately transported to the fish processing facility under regular conditions (storage tank with water temperature around 15 °C), where a standard processing procedure was used (stunning, slaughtering, cleaning, desliming, descaling, gutting, and sectioning into de-skinned fillets with 100 ± 20 g average mass). Good hygiene practice and a high degree of cleaning were taken into consideration. During processing, all abiotic surfaces that came into contact with the carp flesh (scaling table, deskinning table, filleting table, packaging table, scale, package) were sampled and examined for the enumeration of microorganisms with standardized methods (MSZ EN ISO 4833–1 2014). Carp fillets were individually packaged in both AP and VP form and were stored for the expected time of microbial stabilization (96 h) at 2 ± 1 °C and 6 ± 1 °C in both package types for subsequent bacterial analysis. As frozen storage is a common method for preservation of fish products (Ježek and Buchtová 2010), a 120-day-long storage experiment was set at − 18 ± 2 °C with both aerobic package (FAP) and vacuum package (FAV). All settings were performed in triplicates. The microbial community of initial samples, chilled and frozen-thawed AP, and VP samples were all characterized as follows.

### Cultivation-independent microbial characterization

Illumina 16S rRNA amplicon sequencing was used to precisely assess the bacterial community composition of the chosen samples. DNA was extracted with the DNeasy tissue kit (Qiagen, Germany) from 10 to 20 mg tissue samples following the instructions of the manufacturer. For paired-end 16S rRNA amplicon sequencing, the variable V3 and V4 regions of the 16S rRNA gene were amplified by using primers with Illumina adapter overhanging oligonucleotides (Klindworth et al. 2013). Each polymerase chain reaction (PCR) contained 12.5 ng of DNA, 0.2 μmol/L of each Illumina 16S primers, and 12.5 μL of 2X KAPA HiFi HotStart Ready Mix (KAPA Biosystems, London, UK) supplemented with molecular grade water to 25 μL final volume. The PCR temperature profile was the following: initial denaturation for 5 min at 95 °C and 25 cycles of amplification (30 s at 95 °C, 30 s at 55 °C, 30 s at 72 °C). The last step was a final extension for 5 min at 72 °C. All amplifications were carried out in a ProFlex PCR System (Applied Biosystems by Life Technologies, USA). Amplicons were analysed by agarose gel electrophoresis. Paired-end fragment reads were generated on an Illumina MiSeq sequencer using MiSeq Reagent Kit v3 (600-cycle). Read numbers ranged between 68,526 and 94,833. Primary data analysis (base-calling) was carried out with Bcl2fastq^ software (v2.17.1.14, Illumina). Reads were quality- and length-trimmed in CLC Genomics Workbench Tool 9.5.1 using an error probability of 0.05 (Q13) and a minimum length of 50 nucleotides as a threshold. Trimmed sequences were processed using mothur v 1.41.1 (Schloss et al. 2009) as recommended by the MiSeq SOP page (http://www.mothur.org/wiki/MiSeq_SOP) (Kozich et al. 2013). Sequences were assorted based on the alignment using the SILVA 132 SSURef NR99 database (Quast et al. 2013). Chimera detection was performed with mothur’s uchime command (Edgar et al. 2011), and ‘split.abund’ command was also used to remove singleton reads according to Kunin et al. (2010). The standard 97% similarity threshold was used to determine operational taxonomic units (OTUs) as suggested by Tindall et al. (2010) for prokaryotic species delineation. For species-level identification, pair-end sequences were screened for a minimum length of 400 bp (‘screen.seqs’ command), which enables species prediction (Franzén et al. 2015). Raw sequence reads were deposited in the National Center for Biotechnology Information — Sequence Read Archive (NCBI-SRA) under BioProject ID PRJNA613591. The most abundant OTUs were also identified by using the EzBioCloud 16S rRNA gene database (Yoon et al. 2017). Shannon–Weaver diversity indexes were calculated based on 16S amplicon sequencing data by the mothur software.

### Isolation and enumeration of bacterial community

With a robust cultivation approach using the pour plate technique with three different cultivation media, our aim was to compare the effectiveness of commonly used cultivation and 16S rRNA-based identification with the NGS method and to validate NGS to species-level identification of storage microorganisms. Fillets were sampled and examined by standardized microbial methods for enumeration and isolation of microorganisms (ISO 6887–2 2003; ISO 6887–3 2017). A 25-g batch from each sample was homogenized with phosphate-buffered saline (PBS) in a 1:10 ratio. A series of tenfold dilution was prepared in PBS, and all levels of dilutions were plated with a modified tryptone glucose yeast extract (TGY) agar (tryptone, 10 g, glucose 1 g, yeast extract, 10 g, bacteriological agar, 18 g, distilled water, 1 L), a medium recommended for environmental samples and food products, for the determination of colony forming units (cfus) and for the isolation of dominant cultivable bacteria. Incubation parameters were 28 ± 1 °C, 96 h. For selective isolation, additional media were used parallelly. *Acinetobacter* spp. were cultivated on Acinetobacter CHROMagar™ (Paris, France), Aeromonads were grown on Aeromonas Medium (LabM, Neogen, UK). After the incubation period, TGY plates and the applied selective/differential media were visually checked, and the colony forming units (cfu) were determined. Colonies with visible differences in morphology were subcultured for further investigations.

### Bacterial identification

Bacterial strains were priorly identified by matrix-assisted laser desorption ionization time-of-flight (MALDI-TOF) mass spectrometry (MS) method using a Bruker Biotyper system (Bruker Daltonics, Leipzig, Germany). For MALDI-TOF MS identification, bacterial samples were prepared according to the instructions of Bruker Daltonics. Biological material (0.1–0.2 mg) of the examined culture was smeared as a thin film directly onto the spot on a MALDI target plate and was overlaid with 1 μL HCCA (4-hydroxy-α-cinnamic acid) matrix (Bruker Daltonics, Bremen, Germany). Samples were measured automatically by the MALDI Biotyper 3.0; spectra were compared to the Biotyper database and were analysed by flexAnalysis software version 3.3. According to the ranges of score values recommended by the manufacturer (Bruker Daltonics), results were divided into four categories (2.300–3.000: highly probable species identification; 2.000–2.299: secure genus identification, probable species identification; 1.700–1.999: probable genus identification; 0.000–1.699: not reliable identification). If score value was lower than 2.000, or MALDI-TOF MS method failed (no peaks found), partial 16S rRNA sequencing was used for the species-level identification of isolates.

For 16S rRNA gene sequencing, overnight liquid cultures of the isolated strains were extracted and purified using the MOBIO Ultra Clean Microbial DNA Isolation Kit (MOBIO Laboratories, USA) following the instructions of the manufacturer. For amplification of 16S rRNA genes, bacteria-specific universal primers (27 forward and 1492 reverse) were used (Lane 1991). Reaction parameters were as follows: 98 °C for 5 s; 32 cycles of 94 °C for 30 s, 52 °C for 30 s, and 72 °C for 45 s; and final extension at 72 °C for 10 min. The nucleotide sequence determination was performed with the Big Dye Terminator version 3.1. Cycle Sequencing Kit (Applied Biosystems, USA) and sequences were analysed with ABI 3130 Genetic Analyzer (Applied Biosystems, USA). Prior to capillary gel electrophoresis, products were purified by ethanol precipitation. The obtained (> 400 bp) sequences were edited and assembled using MEGA5 software^®^ and were searched for homology in the EzBioCloud database (Yoon et al. 2017). Sequence homology over 98% was accepted as species-level identification. Sequence data obtained in this study were deposited in the National Center for Biotechnology Information (NCBI) GenBank database under the following accession numbers: MT225805–MT225940.

## Results

### Background parameters

At the time of sampling, water samples were dominated by hydrogen-carbonate (over 250 mg/L), while sediment samples had a higher concentration of sulphate (400–800 mg/kg dry mass) and calcium ions (340–511 mg/kg dry mass). Waterbody showed low bacterial abundance (6 × 10^3^ cfu/mL for inflow and 8 × 10^2^ cfu/mL for waterbody); faecal indicator *Escherichia coli* and the opportunistic pathogen *Pseudomonas aeruginosa* were not detectable. During processing, all abiotic surfaces that come into contact with the carp flesh were sampled and examined for the enumeration of microorganisms. The determined cfu values of cultivable microorganisms were as follows: 4 × 10^1^ cfu (scaling table), 4.1 × 10^4^ cfu (deskinning table), 1.2 × 10^4^ cfu (filleting table), 1.8 × 10^3^ cfu (packaging table), 4.1 × 10^2^ cfu (scale), and < 10 cfu (package). Indicator and pathogen microorganisms were not detectable in the processing facility.

### Characterization of the microbial community and cell counts

**Fig. 2:**
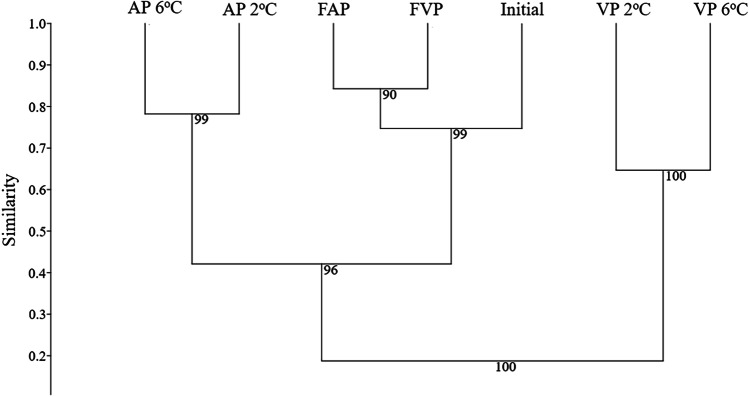
UPGMA dendrogram showing OTU cluster analysis of 16S amplicon sequencing data based on the Bray–Curtis similarity index. Initial — initial fish fillet sample at 0 h of the storage experiment; AP 2 °C — aerobic package stored 96 h at 2 °C; AP 6 °C — aerobic package stored 96 h at 6 °C; VP 2 °C — vacuum package stored 96 h at 2 °C; VP 6 °C — vacuum package stored 96 h at 6 °C; FAP — frozen-thawed aerobic package stored 120 days at − 18 °C; FVP — frozen-thawed vacuum package stored 120 days at − 18 °C

Microbial communities of the initial and the stored carp samples (96 h: AP 2 °C, AP 6 °C, VP 2 °C, VP 6 °C; 120 days: FAP, FAV) were comprehensively characterized by both cultivation-independent (NGS) and cultivation-dependent (MALDI-TOF MS and 16S rRNA sequencing) methods. The OTU cluster analysis, based on the Bray–Curtis similarity index, revealed that the seven examined sample types clustered into three different groups (Fig. 2). The 96-h AP 2 °C and AP 6 °C samples and the initial (0 h), frozen-thawed (120 days) samples formed two distinct clusters, while 96 h VP 2 °C and VP 6 °C samples had more unique community profiles. Based on Shannon indexes, the initial diversity (2.86) decreased during the 2 °C and 6 °C storage, and it was the lowest in the case of chilled AP samples (1.76 at 6 °C and 2.24 at 2 °C), while the highest level of diversity was observed in the frozen-thawed (FAP, FVP) samples (2.94–3.1).

**Fig. 3:**
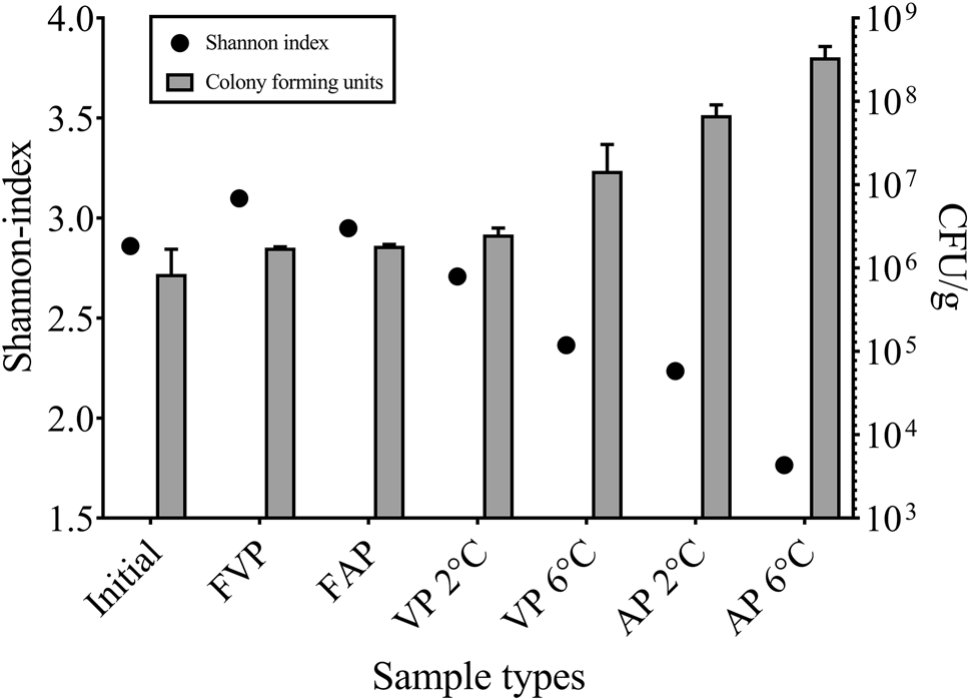
Microbial diversity (Shannon index) of the carp samples indicated by 16S Illumina amplicon sequencing and the average values of colony forming units (cfu) on TGY medium. Initial — initial fish fillet sample at 0 h of the storage experiment; AP 2 °C — aerobic package stored 96 h at 2 °C; AP 6 °C — aerobic package stored 96 h at 6 °C; VP 2 °C — vacuum package stored 96 h at 2 °C; VP 6 °C — vacuum package stored 96 h at 6 °C; FAP — frozen-thawed aerobic package stored 120 days at − 18 °C; FVP — frozen-thawed vacuum package stored 120 days at − 18 °C

Based on the applied cultivation-dependent methods, initial cell counts of the stored fish samples ranged from 2.5 × 10^5^ to 2.1 × 10^6^ cfu/g and showed a steady growth till the end of chilled storage (96 h) on TGY agar. According to the number of cultivable microorganisms on the TGY medium, the vacuum package at 2 °C showed the lowest bacterial cell counts, while the aerated package at 6 °C had the highest cfu values. Based on Pearson’s correlation (*r*) calculated with a confidence interval of 95%, Shannon indexes and colony forming units on TGY agar showed a significant, inverse correlation (*r* = − 0.8399), namely the higher cfu values were associated with lower microbial diversity in the examined samples (Fig. 3).

**Fig. 4:**
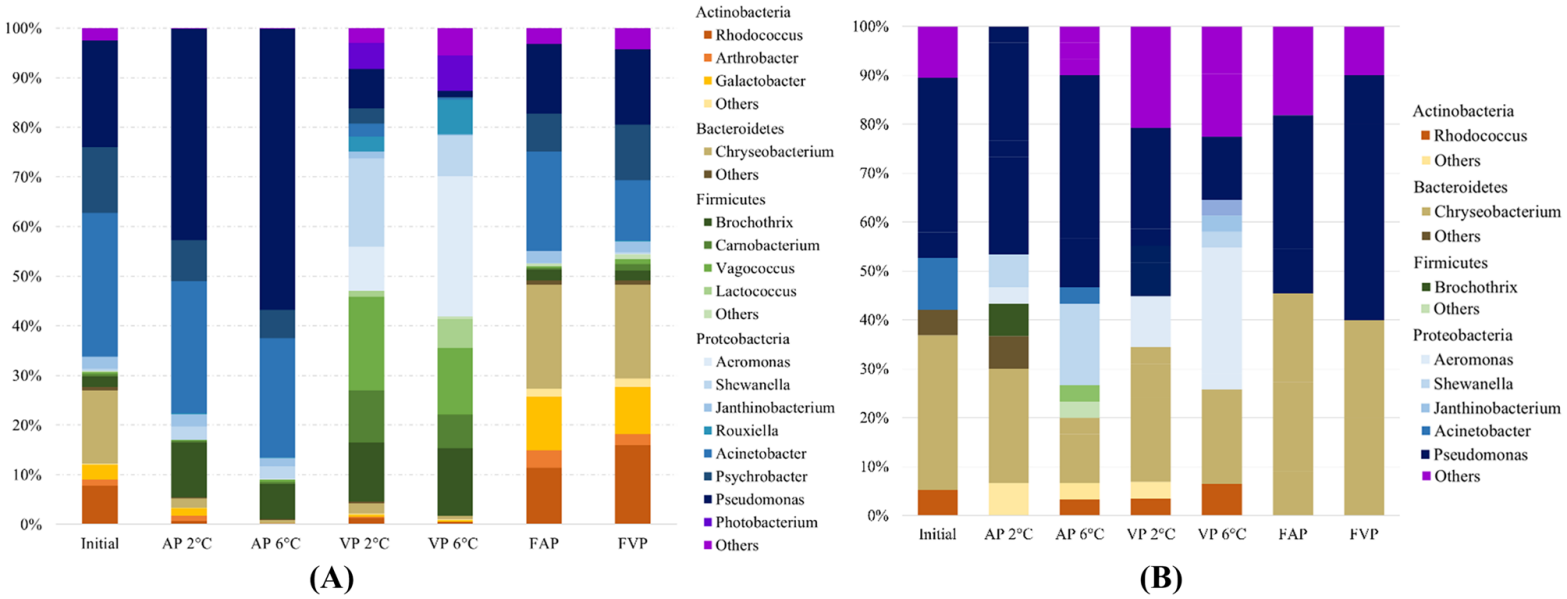
Distribution of the microbial community of common carp samples obtained by Illumina 16S rRNA gene amplicon sequencing (**A**) and cultivation-dependent methods (**B**). Initial — initial fish fillet sample at 0 h of the storage experiment; AP 2 °C — aerobic package stored 96 h at 2 °C; AP 6 °C — aerobic package stored 96 h at 6 °C; VP 2 °C — vacuum package stored 96 h at 2 °C; VP 6 °C — vacuum package stored 96 h at 6 °C; FAP — frozen-thawed aerobic package stored 120 days at − 18 °C; FVP — frozen-thawed vacuum package stored 120 days at − 18 °C

The distribution and the relative abundance of the microbial community revealed by cultivation-independent (NGS) and cultivation-dependent (MALDI-TOF MS, partial 16S rRNA sequencing) methods are summarized in Fig. 4. The applied NGS method proved that members of Proteobacteria (45.5–91%), Firmicutes (3.5–42%), Bacteroidetes (0.6–21.7%), and Actinobacteria (0.3–29.3%) dominated all microbial communities (Fig. 4A). The relative abundance of Proteobacteria species was influenced by the package type: the initial ratio (69%) increased in the AP 2 °C and 6 °C samples to 83–91% during storage, while it decreased in both VP (52–57%) and frozen-thawed (45–47%) samples. The abundance of Firmicutes phylum was low in the initial and frozen-thawed samples (3.2–5.4%), slightly increased in the AP samples (7.9–11.6%) and became substantial in the VP samples (42–39.6%). At lower temperatures, a slightly higher Firmicute ratio was observed in both VP and AP samples. Bacteroidetes were abundant in the initial (15.5%) and frozen-thawed samples (19.7–21.7%) but not in the chilled AP and VP samples (0.6–2.5%). The Actinobacteria phylum showed the same distribution as the Bacteroidetes: the most abundant genera (*Rhodococcus*, *Galactobacter*, and *Arthrobacter*) had a relatively high abundance in the initial and frozen-thawed samples but decreased in AP and VP samples.

Based on cultivation-dependent identification results, Bacteroidetes and Proteobacteria phyla were isolable from all samples, while Actinobacteria and Firmicutes members were only marginally detectable (see Fig. 4B).

## Species-level identification of the microbial community

The 16S rRNA gene sequences obtained from NGS data and the partial 16S rRNA sequences of the cultivated isolates had at least 400 bp length and showed a 98.2–100% similarity with the reference sequences of the EzBioCloud database (Yoon et al. 2017). According to Franzén et al. (2015), the applied sequence lengths and similarity levels enabled a species-level prediction to reveal the dominant species of carp fillet samples. The species-level identification of the most abundant 20 OTUs obtained by NGS and their cultivable counterparts is summarized in Table 1.

Within Bacteroidetes, the dominance of the *Chryseobacterium* species was observable. Based on our NGS dataset, three species (*C. ginsengiterrae*, *C. piscium*, and *C. shigense*) were abundant in the initial and frozen-thawed samples (2.0–7.5%). Among Actinobacteria, *Rhodococcus erythropolis* and *Galactobacter caseinivorans* were the most abundant species; both were detectable in the initial (3%) and the frozen-thawed flesh samples (2.9–15.2%). Bacteroidetes and Actinobacteria species significantly decreased during all types of chilled storage. Among Firmicutes, *Brochothrix thermosphacta*, a common fish spoilage organism (Stanborough et al. 2017), was detectable in the initial and frozen-thawed samples and became one of the most abundant species in AP and VP samples (11.2–13.5%). Similarly, *Carnobacterium maltaromaticum* (formerly *C. piscicola*) dominated the microbial communities of VP samples (6.6–10.4%). Another Firmicutes, *Vagococcus vulneris*, a Gram-positive, catalase-negative, coccus-shaped, motile, lactic bacterium, which was recently described as a new species isolated from a human foot wound (Shewmaker et al. 2019), showed abundance (13.2–18.7%) in VP samples.

Proteobacteria species were highly abundant in all examined sample types. Pseudomonads, such as *P. psychrophila* and *P. azotoformans*, were found in large numbers. The initial abundance (12.7%) of *P. psychrophila* increased in the AP samples (32.7–51.8%), while it was less significant in the VP and frozen-thawed samples (6.7–0.8%). *P. azotoformans*, showed a similar distribution, but elevated temperature (6 °C) had a negative effect on its growth. Among Acinetobacters, our study revealed that the most abundant OTU in the initial sample (14.3%) has a 99.36% similarity to *A. albensis.* The initial ratio of this genotype decreased in both AP and VP samples during storage. The second most abundant species from the Acinetobacter genus was *A. harbinensis*, a species recently described from a river in China (Li et al. 2014). According to our results, the initial abundance (7.9%) of *A. harbinensis* almost doubled in both AP samples (14.1–15.1%), but vacuum package and freezing were not favourable for the growth of this bacterium. Another frequent (4.8%) genotype of the initial sample showed a 97.9% homology with the psychrotolerant species *A. celticus*, previously isolated from natural soil and water samples (Krizova et al. 2016); however, the determined sequence homology with the *A. celticus* type strain was low. Among aeromonads, *Aeromonas sobria* species complex was detected by NGS in VP samples (8.8–27.9%). Among Psychrobacters, *P. cibarius* and *P. phenylpyruvicus* were represented in the initial samples (5.3–7.9%). *P. cibarius* remained abundant in AP samples (5.4–7.1%), while the ratio of *P. phenylpyruvicus* decreased (0.32–1.15%). *Shewanella baltica*, a known opportunistic microbial pathogen of humans and aquatic animals, which is often linked to the spoilage of chilled food products (Beaz-Hidalgo et al. 2014), was not abundant in the initial sample (0.3%) but slightly increased in the AP samples (2.3–2.5%) and became important in the VP 2 °C (7.5%) and especially in the VP 6 °C sample (15%). Among Photobacterium species, *P. carnosum* was identified in the VP samples (5.2–6.9%), but not in the AP samples. *Janthinobacterium lividum* was detectable in the initial and AP samples stored at 2 °C (2.4%), and frozen-thawed storage did not affect its abundance. At the same time, it was less abundant in samples stored at 6 °C or in VP (1.6–0.2%). In VP samples, a facultative anaerobe representative of a new genus within Proteobacteria, *Rouxiella chamberiensis*, became an abundant species (3.0–6.8%) which was firstly isolated and described from parental nutrition bags (Le Flèche-Matéos et al. 2015).

Parallelly with NGS, 220 strains were isolated with cultivation-dependent methods, of which 161 cultures were successfully identified at the species level, based on partial 16S rRNA gene sequence homology and/or MALDI-TOF MS. The identified isolates belonged to 52 different species. Dominant species were the members of *Pseudomonas synxantha/mucidolens/azotoformans/libanensis/lactis/paralactis* group (21.1%), *Chryseobacterium ginsengiterrae* (13.6%), *Pantoea agglomerans* (6.1%), and *Chryseobacterium piscium* (5.5%). The complete list of the isolated and identified isolates and the detailed results of cultivation and identification processes are summarized in Supplementary materials (S1–S3). Despite the application of special cultivation media, nine of the most abundant 20 species were not cultivable. At the same time, several *Aeromonas* species were cultivable from vacuum packed fillets, such as *A. veronii/ichtiosmia*, *A. media/rivipollensis*, *A. salmonicida/bestiarum/popoffii*, and *A. encheleia*, but similarly to NGS results, the discriminatory power of partial 16S rRNA sequencing did not enable a reliable species-level identification of Aeromonads.

## Discussion

The microbial composition of freshwater fish products is still in great interest to increase the shelf-life of raw products. Hungary is the 3rd largest producer of common carp (*Cyprinus carpio*) in Europe (FAO 2021) and a good representative of the regional climatic and production conditions. Our aim was to evaluate the initial microbiological state of carp fillets and their changes under different storage and package conditions and to identify new members of stored carp fillets with the parallel application of NGS and cultivation-dependent methods. Comparing to scientific literature, the determined cfu values of the initial carp fillets were unexpectedly high, only slightly below the initial contamination level for raw fish immediately after production (Stannard 1997) but did not reach the maximum microbial load limit of freshwater fish (Odeyemi et al. 2020). The possible sources of this initial microbial load of the flesh are the skin, gills, intestine, fish processing technology, or environmental contamination (Sterniša et al. 2016). Since the examined aquaculture and the abiotic surfaces during processing showed low cfu values, the high initial cell counts of fillets can be attributed to a cross-contamination originating from the skin, gills, or intestine occurring during temporary storage or processing. Microbial rejection point, determined as 10^7^ cfu/g (ICMSF 1986), was reached after 96 h in AP 2 °C, AP 6 °C, and VP 2 °C samples, but microbial levels stayed lower than the usual cell counts at the moment of sensory rejection determined as 10^9^ cfu/g (Kuuliala et al. 2018). During storage, the increasing bacterial numbers paired with a loss of diversity due to a subset of bacteria that became dominant are in accordance with a recent study on refrigerated pork (Li et al. 2019). Based on cluster analysis, the effect of temperature and package type on the microbial community of carp fillets was supplemented with new information. In a recent study, Pan et al. (2018) verified that the microbial communities of stored crips grass carp fillets do not cluster based on packaging. Our results support that in the initial phase of common carp storage, the package type is a determining factor in clustering, while temperature affects the bacterial cell counts.

The revealed composition of microbial communities with the dominance of Proteobacteria, Firmicutes, Bacteroidetes, and Actinobacteria is in accordance with previous investigations (Wang et al. 2017; Li et al. 2018) using 16S rRNA gene identification of bacterial isolates obtained from chilled carp fillets, but Firmicutes did not reach the overwhelming dominance reported previously after 12-day-long storage (Zhang et al. 2015). The microbial composition of the examined carp samples revealed previously unknown members of raw flesh microbiota. Eight out of the top twenty species identified by NGS have never been linked to raw carp fillet samples, and their direct or indirect effects on spoilage are unknown.

Among Bacteroidetes, little is known about the identified *Chryseobacterium* species (*C. ginsengiterrae*, *C. piscium*, and *C. shigense*) found in the initial and frozen-thawed samples. The type strain (LMG 23,089) of *C. piscium* was previously isolated from fresh fish caught in the South Atlantic Ocean (de Beer et al. 2006). *C. shigense* was detected from a beverage in Japan (Shimomura et al. 2005) and was isolated from farmed rainbow trout during disease outbreaks (Zamora et al. 2012). *C. ginsengiterrae* was recently isolated from a ginseng field (Noh et al. 2017) and has not been previously identified in raw food products. Based on scientific sources, psychrotolerant *Chryseobacterium* species can cause spoilage defects due to their proteolytic and lipolytic activity as it was described in raw milk samples (Yuan et al. 2018) but, considering their low numbers in 96 h AP and VP samples, the effect of the identified species on the quality of stored carp seems to be negligible.

Similarly, the role of the identified Actinobacteria, *R. erythropolis*, and *G. caseinivorans*, in chilled storage or food spoilage, has not been specified previously in scientific literature. *G. caseinivorans* needs further attention since it was recently described from bulk tank raw cow’s milk from three different dairy farms in Germany (Hahne et al. 2019b), which underlines its importance in raw animal food products.

Among Firmicutes, the potential contributor to carp spoilage under the examined package and temperature conditions was *Brochothrix thermosphacta*, a fermentative bacterium. *B. thermosphacta* can be dominant in vacuum-packed food products (Nowak et al. 2012), produces organoleptically unpleasant compounds, and its role in shortening the shelf-life of food makes it significant in aerobic storage as well. *C. maltaromaticum*, a commonly detectable species in cold environments and chilled food, such as meat, seafood, and dairy products (Leisner et al. 2007), was another abundant species in vacuum packages, which is in accordance with previous reports of modified-atmosphere-packed food (Paludan-Müller et al. 1998; Paarup et al. 2002). However, the volatile molecules produced by this species have low sensory impacts (Casaburi et al. 2011). There is no available information of the newly detected storage organism *Vagococcus vulneris*, but the closely related *V. salmoninarum* is a verified producer of 2-heptanone, 2-nonanone, and 2-undecanone (Calliauw et al. 2016). In the future, *V. vulneris* should be cultivated, identified with complete 16S rRNA sequencing, and its spoilage potential should be examined to clarify its effects on sensory characteristics.

Among Proteobacteria, *Pseudomonas*, *Acinetobacter*, and *Psychrobacter* genera were the most abundant in the initial raw fillets following the previous findings on stored silver carp (Jia et al. 2018). Regarding Pseudomonads, there is a debate in the scientific literature on *P. lindens*, *P. fluorescens*, *P. putida*, and *P. fragi/psychrophila*, as the dominant members of the microbial community in chilled meat and fish (Wickramasinghe et al. 2019; Gennari and Dragotto 1992). Our results emphasize the importance of *P. psychrophila*, a species that can form a substantial amount of biofilm during chilled storage, providing favourable conditions for the growth and persistence of pathogenic *E. coli* (Sterniša et al. 2019). Based on our results, the overwhelming dominance of *P. psychrophila* is verified in AP samples, while its abundance in VP and frozen-thawed samples was lower following the findings of Tryfinopoulou et al. (2002).

*Acinetobacter* spp. are typical members of the psychrophilic spoilage microbiota of chilled foods (Betts 2006; Li et al. 2017b) and were represented in our dataset, too. *A. harbinensis* was previously detected in silver carp fillets stored at 4 °C (Jia et al. 2018), but *A. albensis*, a species recently isolated and described from a well-protected landscape area in the Czech Republic (Krizova et al. 2015), was just proved to be predominant in raw milk samples (Hahne et al. 2019a) and had no link to fish products. While these uncommon species were detectable, *A. johnsonii*, a frequent species in fish and fish products (Zhang et al. 2015), or *A. lwoffii*, a predominant species of spoiled food (Kampfer 2000), were not abundant in our NGS dataset, and only one *A. johnsonii* strain was cultivable. According to metagenomic studies, the possible source of the detected *Acinetobacter* species is the gut microbiota of carp (Hovda et al. 2007; Etyemez and Balcázar 2015). Regarding Aeromonads, the occurrence of *A. sobria* species complex was in accordance with data of stored and refrigerated foods such as raw fish (Alhazmi 2015) and vacuum-packed milkfish (Simon et al. 2016). However, considering the low discriminatory power of 16S rRNA for the genera *Acinetobacter* (Alvarez-Buylla et al. 2012) and *Aeromonas* (Miñana-Galbis 2009), additional identification methods are recommended in the future to reach a reliable differentiation at the species level. Regarding Psychrobacters, the available information on the identified species supports their minor role in spoilage defects. *P. cibarius* does not produce a significant amount of volatile organic compounds at 4 °C, just a slight amount of trimethylamine (TMA) (Broekaert et al. 2013). Regarding *P. phenylpyruvicus*, our findings agree with previous reports describing its decrease at the latter stage of cold storage of freshwater fish (González et al. 2000). The identified *Shewanella* species, *S. baltica*, may play a significant role in spoilage since it is the most important H_2_S-producing bacterium in ice-stored marine fish (Vogel et al. 2005) with a possible role in modifying texture and sensory characteristics (Serio et al. 2014). The detected *Photobacterium* species, *P. carnosum*, was abundant in 96 h VP samples following previous findings of its adaptability to modified-atmosphere environments (Hilgarth et al. 2018; Fuertez-Perez et al. 2019). *Photobacteria* produce biogenic amines and other spoilage compounds (Takahashi et al. 2015; Höll et al. 2019), contributing to the spoilage of modified air products. *Janthinobacterium* is a basic group of marine fish gut microbiota (Egerton et al. 2018) with the ability to produce exopolysaccharide-rich biofilms (Pantanella et al. 2007), but the identified species, *J. lividum*, has not been reported previously in stored fish fillets. Similarly, the identified *Rouxiella* species, *R. chamberiensis*, was recently proved to be a member of salmon gut microbiota (Higuera-Llantén et al. 2018), but our result is the first verification of its presence in carp products. According to scientific literature, the possible source of *J. lividum* and *R. chamberiensis* is the carp gut microbiota and their possible role in spoilage should be characterized in the future.

## Conclusions

This paper aims to improve the understanding and knowledge of the storage microbiota of carp fillet originated from a European polyculture with low population density. High initial cell counts emphasize that under the given climatic and production conditions (e.g. higher water temperatures in aquaculture and during temporary storage, higher initial microbial load of gills, integument, or in the digestion system), microbial rejection point can be reached rapidly if package and storage conditions are not optimal. Our results verified that in the initial phase of chilled storage of common carp, the package type is a determining factor in clustering, while temperature primarily affects the bacterial cell counts. To increase shelf-life, the initial microbial load of raw carp fillets should be decreased with the close monitoring of transport, processing, and storage conditions, whenever possible. The recommended storage of freshwater carp fillets is the vacuum package at 2 °C. Based on our NGS results, the microbial composition of the examined initial and stored common carp samples is slightly different from the previously described microbiotas. Cultivation-dependent identification methods revealed only 45.0% of the most abundant 20 OTUs identified by NGS. Since the applied species-level identification methods have their limitations, a complete 16S rRNA sequence analysis is required in the future to verify the species-level identification of the detected genotypes. Our results suggest that under the local climatic and production conditions, several uncommon, presumably fish gut or environmentally transmitted bacteria are abundant during the initial storage of common carp. The newly detected members of carp fillet microbiota, such as *Vagococcus vulneris*, *Rouxiella chamberiensis*, and *Acinetobacter albensis*, have a still unknown role in spoilage and shelf-life. In the future, knowing the members of the initial microbial community of flesh enables the optimization of processing and storage technology to reach a high level of compliance with food safety requirements that are significant for the development of production and storage.

**Table 1:**
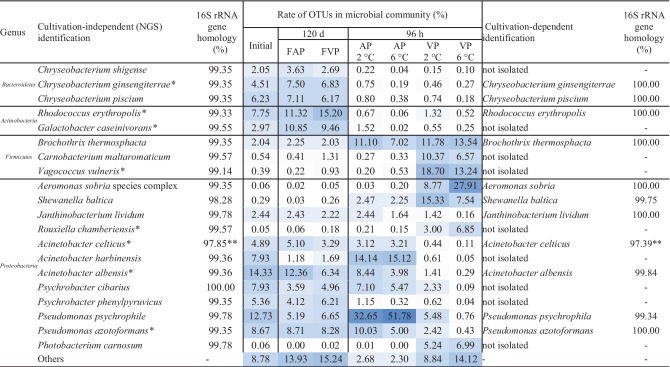
The most abundant OTUs of common carp fillets and their cultivable counterparts

Blue colour — relative abundance (dark — high-abundance OTUs); initial — initial fish fillet sample at 0 h of the storage experiment; AP 2 °C — aerobic package stored 96 h at 2 °C; AP 6 °C — aerobic package stored 96 h at 6 °C; VP 2 °C — vacuum package stored 96 h at 2 °C; VP 6 °C — vacuum package stored 96 h at 6 °C; FAP — frozen-thawed aerobic package stored 120 days at − 18 °C; FVP — frozen-thawed vacuum package stored 120 days at − 18 °C

*Previously unknown contributors in carp storage

**Low homology with type strain

## Supplementary Information

Below is the link to the electronic supplementary material.

Supplementary file1 (CSV 9 KB)

Supplementary file2 (XLSX 172 KB)

Supplementary file3 (PDF 976 KB)

## Data Availability

Data generated or analysed during this study are included in this published article [and its supplementary information files], or available from the corresponding author on reasonable request.

## Abbreviations

AP: Air-packed
CFU: Colony forming unit
FAP: Frozen-thawed aerobic package
FAV: Frozen-thawed vacuum package
LAB: Lactic acid bacteria
MALDI-TOF: Matrix-assisted laser desorption ionization time-of-flight
MPN: Most probable number
MS: Mass spectrometry
NCBI-SRA: National Center for Biotechnology Information — Sequence Read Archive
NGS: Next-generation sequencing
OTU: Operational taxonomic unit
PBS: Phosphate buffered saline
PCR: Polymerase chain reaction
SSO: Specific spoilage organisms
TGY agar: Tryptone, glucose, yeast extract agar
TMA: Trimethylamine
T-RFLP: Terminal restriction fragment length polymorphism
VP: Vacuum-packed
