# Supplementary figures and images for: Novel members of bacterial community during a short-term chilled storage of common carp (Cyprinus carpio)
Source: Folia Microbiol (Praha). 2021 Dec 7;67(2):299–310. doi: 10.1007/s12223-021-00935-4 (PMC8933370; doi:10.1007/s12223-021-00935-4)

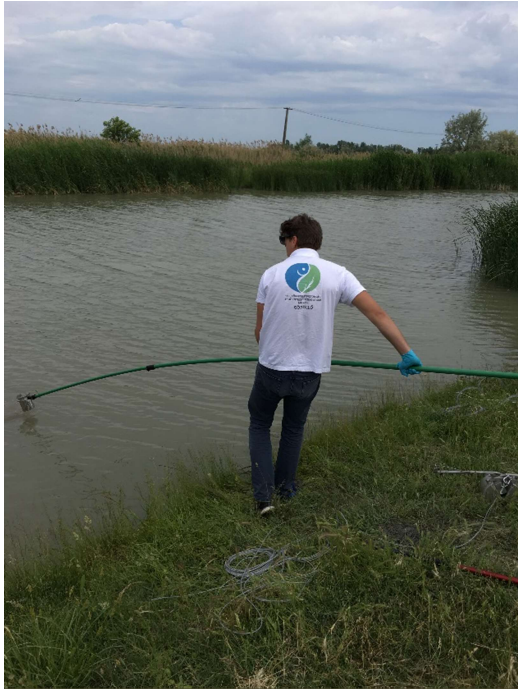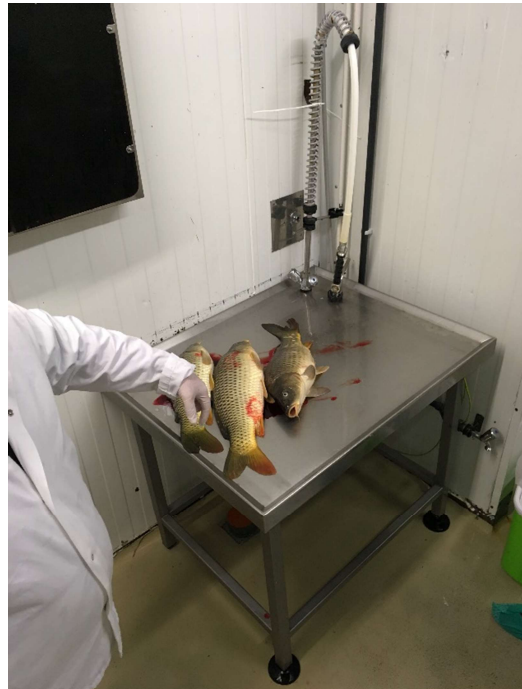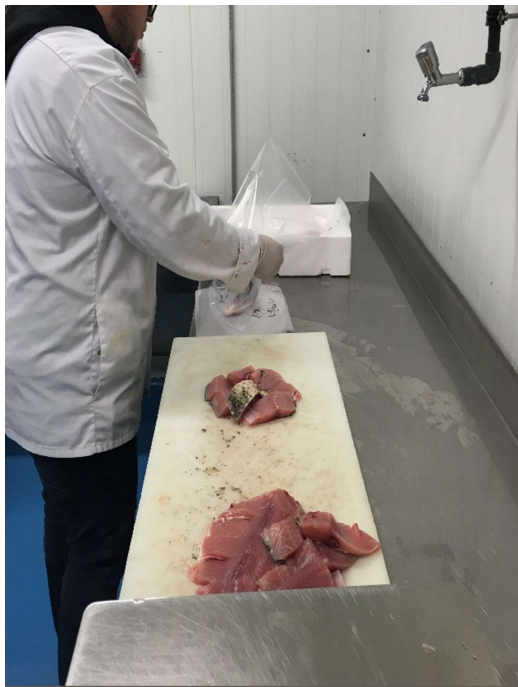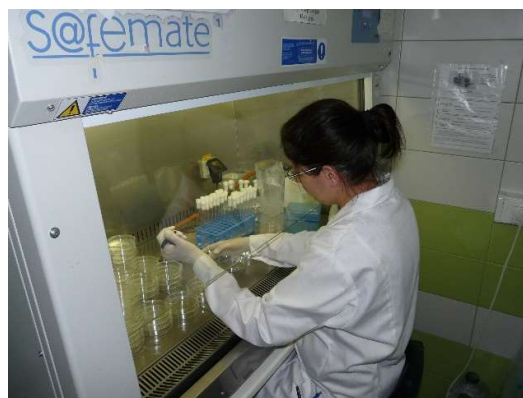

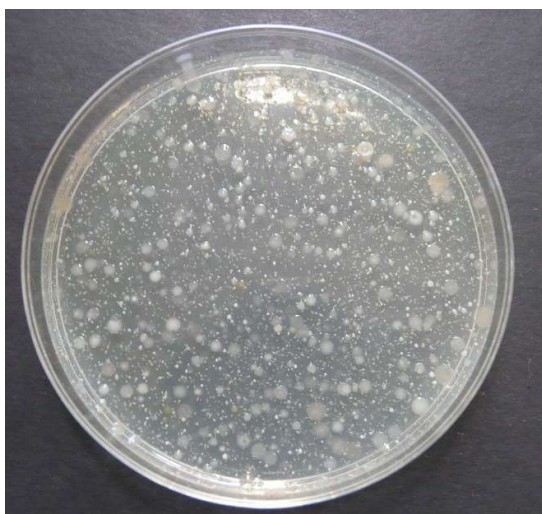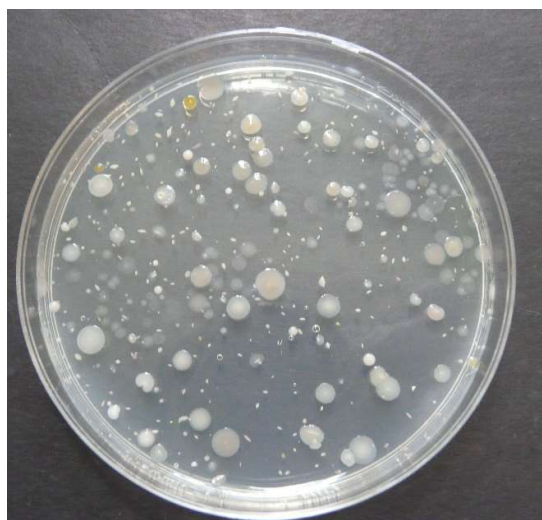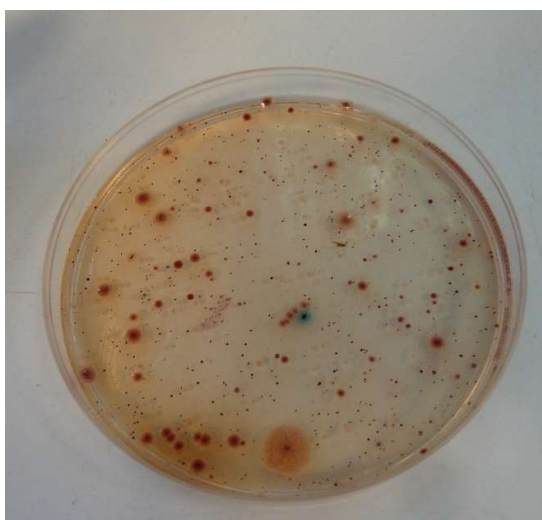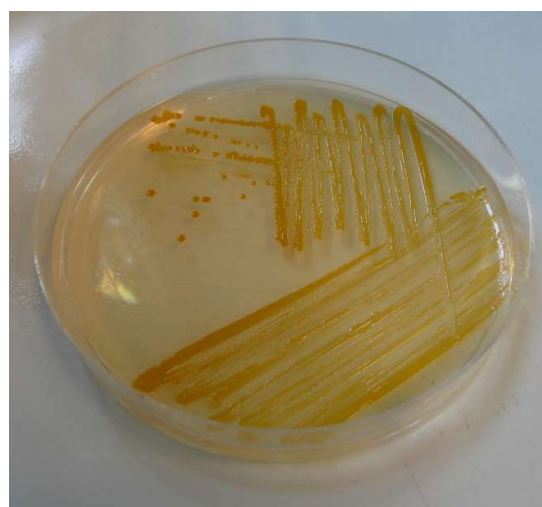

Supplement: Supplementary file 3 — Supplementary file3 (PDF 976 KB) [file 12223_2021_935_MOESM3_ESM.pdf]
